# Simulating My Own or Others Action Plans? – Motor Representations, Not Visual Representations Are Recalled in Motor Memory

**DOI:** 10.1371/journal.pone.0084662

**Published:** 2013-12-18

**Authors:** Christian Seegelke, Charmayne Mary Lee Hughes, Thomas Schack

**Affiliations:** 1 Neurocognition and Action Research Group, Faculty of Psychology and Sport Sciences, Bielefeld University, Bielefeld, Germany; 2 Research Institute for Cognition and Robotics (CoR-Lab), Bielefeld, Germany; 3 Center of Excellence Cognitive Interaction Technology (CITEC), Bielefeld, Germany; 4 Institute of Movement Science, Department of Sport and Health Science, Technical University of Munich, Munich, Germany; University of Milan, Italy

## Abstract

Action plans are not generated from scratch for each movement, but features of recently generated plans are recalled for subsequent movements. This study investigated whether the observation of an action is sufficient to trigger plan recall processes. Participant dyads performed an object manipulation task in which one participant transported a plunger from an outer platform to a center platform of different heights (first move). Subsequently, either the same (intra-individual task condition) or the other participant (inter-individual task condition) returned the plunger to the outer platform (return moves). Grasp heights were inversely related to center target height and similar irrespective of direction (first vs. return move) and task condition (intra- vs. inter-individual). Moreover, participants' return move grasp heights were highly correlated with their own, but not with their partners' first move grasp heights. Our findings provide evidence that a simulated action plan resembles a plan of how the observer would execute that action (based on a motor representation) rather than a plan of the actually observed action (based on a visual representation).

## Introduction

A large corpus of work demonstrates that action plans are not generated from scratch for each movement, but features of recently generated plans are recalled and used for subsequent actions [1–7]. For example, participants in Cohen and Rosenbaum [1] reached out and grasped a plunger to move it from a home position (located at a fixed height) to one of five target positions located at different heights. The higher the target position, the lower the participants initially grasped the plunger (and vice versa), indicating that participants anticipated and planned their actions based on future task demands (i.e., the height of the target) and did so that their limbs would be placed in comfortable or controllable body postures at the end of the movement. Furthermore, when participants returned the plunger to the home position they grasped the plunger close to where they had grasped it before. Cohen and Rosenbaum [1] argued that if participants would have generated a new action plan for the return moves, the object should have been grasped at a similar height regardless of target height (as the home platform was located at a fixed height). However, given that grasp heights for the return moves were similar to those of the first moves, they postulated that participants created a new action plan for the first move and then recalled and slightly modified this plan for the return moves in order to reduce the cognitive costs associated with action planning.

There is also a corpus of evidence suggesting that action plans used in action observation are equivalent to that used in action execution [8], and that observing an action triggers internal action simulation processes [9,10], and facilitates the production of similar actions shortly after observation [11–13]. For example, participants in Castiello et al. [12] observed a grasping action made towards a large or small object and then performed a grasping movement to either object. Overall, reach components were faster (e.g., time to peak velocity) and grasp aperture values were smaller when the observed and self-executed actions were directed to the same object (e.g., small observed object and small grasped object), indicating that the observation of an action primed the forthcoming execution of a similar action. Complementing these findings, a number of neuroimaging studies have demonstrated that similar brain regions are activated during both action observation and action execution [14–17], lending further support to the hypothesis that observing another person’s actions activates the corresponding representations in the observer’s motor system by internally simulating the actions.

The first question we addressed in the present experiment was whether the observation of an action is sufficient to elicit plan recall processes. Given the work demonstrating that the visual representation of the observed action is mapped onto a motor representation of the same action during action observation [8], it stands to reason that an observer should be able to recall a simulated action plan and re-use it for their forthcoming executed actions. To this end, we modified the sequential grasping and placing task introduced by Cohen and Rosenbaum [1] to a social interaction scenario. In the intra-individual task, a single participant performed the entire sequence (i.e., first and return moves) while another participant observed the action. In the inter-individual task, one participant performed the first moves (while the other participant observed the action), and the other participant (the partner) carried out the return move. If the observation of an action is sufficient to elicit plan recall processes, we expected that grasp heights should be similar regardless of task condition (intra- vs. inter-individual).

With the second question we aimed at examining what a simulated action plan ‘looks’ like. Specifically, given that it is the observer’s motor representation that is activated it should follow that the simulated action plan should resemble a plan of how the observer would execute that action (i.e., based on the motor representation) rather than a plan of the actually observed action (i.e., based on the visual representation). In support of this view, previous studies have shown greater activity in a simulation circuit during the observation of familiar as well as (physically) learned actions [18–20] suggesting experience dependent influences on action simulation [21,22]. Previous studies mostly used highly stereotyped actions such finger tapping [15] or grasping and object [11–13], and thus, observed and self-executed actions were performed virtually identical. In the present sequential object manipulation task, the same action goal (i.e., placing the object onto a specific platform) could be achieved by different means (i.e., exact grasp height at the object), and previous research has regularly demonstrated the presence of individual differences in those tasks [2,23–26]. Consequently, such a task allows us to dissociate between whether a simulated action plan is based on a visual representation or a motor representation. If action simulation is based on the specific motor representation in the observer, we expected that participants’ grasp height of the return moves should be more similar to their own first move grasp height compared to their partners first move grasp height.

## Methods

### Ethics Statement

The methodology and consent form for this study were approved by the ethics committee at Bielefeld University, and conformed to the declaration of Helsinki. All participants gave their informed written consent to participate in the study

### Participants

12 dyads (mean age = 26.08, SD = 3.68, 7 male, 17 female) participated in exchange for 5€. All participants were right handed, as assessed using the Revised Edinburgh Handedness Inventory [27], had normal or correct to normal vision, and were physically and neurologically healthy. 

### Apparatus

**Figure 1:**
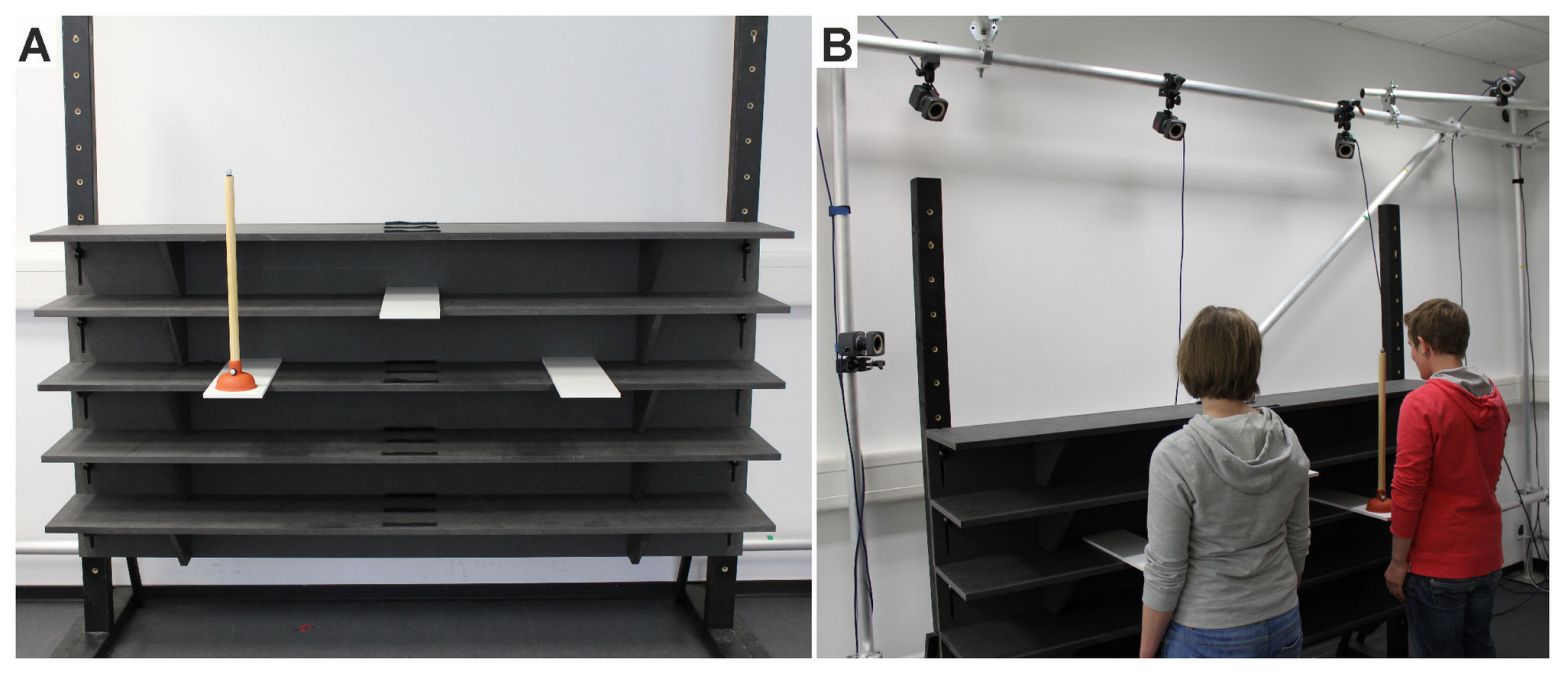
Experimental setup. The participants shown here have given written informed consent, as outlined in the PLOS consent form, to publication of their photographs.

The custom built shelving unit (200 cm x 30 cm) was braced by two legs (Figure 1A). Within the unit, five shelves were located at 50 cm, 70 cm, 90 cm, 110 cm, and 130 cm height. On the 90 cm shelf two outer platforms (45 cm x 15 cm) were positioned 45 cm to either side of the shelving unit midpoint, and extended 15 cm from the shelf. Another platform (45 cm x 15 cm) could be attached to the center of each of the five shelves and served as center platform. The manipulated object was a plunger that had a wooden cylindrical shaft (50 cm in height, 2.5 cm in diameter) and a circular rubber base (5 cm in height, 10 cm in diameter).

Kinematic data was recorded from a retro-reflective marker (10 mm in diameter) placed on the styloid process of the radius (WRT) on the right hand of each participant, and the base of the plunger shaft (PB). Data was recorded at 200 Hz using a ten-camera optical motion capture system (VICON Motion Systems, Oxford, UK). 

### Procedure and design

Participants were arbitrarily designated A and B. Participants stood side by side in front of the outer platforms at a distance of 90 cm (i.e., one in front of the left platform and the other in front of the right platform) and 30 cm from the front edge of the shelving unit (see Figure 1B). At the start of each trial, participants closed their eyes. The experimenter then attached the center platform at the appropriate shelf height, and placed the plunger on the required platform, depending on task condition.

For the intra- and inter-individual task condition, the plunger was initially placed on one of the outer platforms (left or right). After the experimenter verbally indicated which of the two participants would perform the first move both participants opened their eyes, and the named participant grasped and transported the object with the right hand from the outer platform to the center platform (first moves, outer-to-center) and then placed the hand back to the side of the body. The other participant observed the action as it was being performed. The experimenter then verbally stated which participant would perform the return move (center-to-outer).That participant then grasped and transported the object back to the outer platform. Participants operated only in their workspace. Thus, if the plunger was initially located on the left outer platform the participant standing on the left performed the first move. Similarly, if the participant on the right was to perform the return move he or she transported the plunger to the right outer platform. Thus, in the intra-individual task, a single participant (e.g., A) performed both the first and the return move, while the other participant (e.g., B) watched the action as it was performed. In the inter-individual task, one participant (e.g., A) performed the first move while the other participant (e.g., B) watched, and then the other participant (e.g., B) performed the return move.

We also included a control task condition in which we reversed the temporal order of variable to fixed platform heights to control for the possibility that participants would assign greater priority to control at the variable than the fixed platform positions [1].Thus, the plunger was initially placed on one of the five center platforms. After the experimenter verbally indicated which of the two participants would perform the first move both participants opened their eyes, and the named participant grasped and transported the object with the right hand from the center platform to the center platform located on his/ her side, and then placed the hand back to the side of the body (center-to-outer).The other participant observed the action as it was being performed. The experimenter then verbally stated which participant would perform the return move (outer-to-center; this was always the participant who performed the first move in this task).That participant then grasped and transported the object back to the center platform. 

For all task conditions, participants were instructed to perform the movements at a comfortable speed, and to grasp the plunger so that it would not slip through their fingers during the transport. There were a total of 20 trials per participant and each task (inter- vs. intra-individual vs. control), which consisted of each possible combination of center shelf height (50 cm, 70 cm, 90 cm, 110 cm, and 130 cm), and object position (left, right). Participants performed two successive trials per condition, which were presented in a randomized order. The task conditions (intra-individual, inter-individual, control) were performed in a blocked fashion and the order of blocks was counterbalanced across participants. Within each task condition block, participants standing position (i.e., in front of the left or the right outer platform) was balanced. The entire experiment took approximately 45 minutes.

### Data recording and data analysis

The 3D coordinates of the markers were reconstructed and missing data (those with fewer than 10 frames) were interpolated using a cubic spline. The marker coordinates were filtered using a Woltring filter [28] with a predicted mean square error value of 5mm^2^ (Vicon Nexus 1.7). All kinematic variables were calculated using custom written MatLab scripts (Mathworks, Version 7.0). Grasp height was calculated as the vertical distance between WRT and PB, and were extracted from the first frame where the object was grasped from the outer platform. Thus, for the intra- and inter-individual task conditions, grasp heights were extracted at the start of the first moves (outer-to-center) and at the end of the return moves (center-to-outer). In contrast, for the control task condition, grasp heights were extracted at the end of the first moves (center-to-outer) and at the start of return moves (outer-to-center). 

## Results

Grasp height data were analyzed using a 3 task condition (intra-individual, inter-individual, control) × 2 direction (outer-to-center, center-to-outer) × 2 object position (left, right) × 5 center shelf height (50 cm, 70 cm, 90 cm, 110 cm, 130 cm) repeated measures analysis of variance (RM ANOVA). Given that gender can affect cooperation (see 29 for a recent review) and our sample was comprised of more females than males, all analyses were also conducted including only female participants. Analyses revealed the same pattern of results indicating that gender did not affect performance in the present task. Analysis revealed a significant main effect of center shelf height, F(4,92) = 152.61, p < .001, η^2^_p_ = .87 and a significant task condition × direction × center shelf height interaction, F(8,184) = 3.68, p = 0.007, η^2^_p_ = 0.138. To decompose the three-way interaction, additional RM ANOVAs were conducted to directly compare the different task conditions. To control for family-wise errors rate, a Bonferroni correction was applied (α = 0.017). The results are shown in Table 1.

**Table 1:** Results of the three repeated measures ANOVAs (intra-individual vs. inter-individual, intra-individual vs. control, inter-individual vs. control) using the within-subject factors task condition (task), direction (dir), object position (pos), and center shelf height (sh), α = 0.017.

	**intra vs. inter**				**intra vs. control**				**inter vs. control**		
**Variable**	**F**	**p**	**η^2^_p_**		**F**	**p**	**η^2^_p_**		**F**	**p**	**η^2^_p_**
task	0.35	.560	.02		0.01	.935	.00		0.26	.613	.01
dir	0.00	.985	.00		3.29	.083	.13		1.39	.251	.06
pos	0.68	.418	.03		0.04	.841	.00		0.33	.573	.01
sh	140.86	<.001	.86		125.77	<.001	.85		114.18	<.001	.83
task × dir	0.10	.754	.00		1.75	.199	.07		1.74	.200	.07
task × pos	0.10	.750	.00		1.69	.207	.07		2.43	.133	.10
task × sh	1.02	.385	.04		0.81	.470	.03		0.05	.981	.00
dir × pos	2.13	.158	.09		7.64	.011	.25		5.60	.027	.20
dir x sh	0.96	.402	.04		1.74	.189	.07		1.60	.182	.07
pos × sh	0.60	.661	.03		0.16	.960	.01		0.21	.931	.01
task × dir × pos	0.29	.596	.01		1.52	.230	.06		0.44	.513	.02
task × dir × sh	0.43	.720	.02		8.71	.001	.28		4.38	.003	.16
task × pos × sh	0.20	.938	.01		0.93	.450	.04		0.55	.702	.02
dir× pos × sh	0.73	.546	.03		0.31	.828	.01		0.48	.694	.02
task × dir × pos × sh	0.32	.863	.01		0.92	.421	.04		0.52	.718	.02

**Figure 2:**
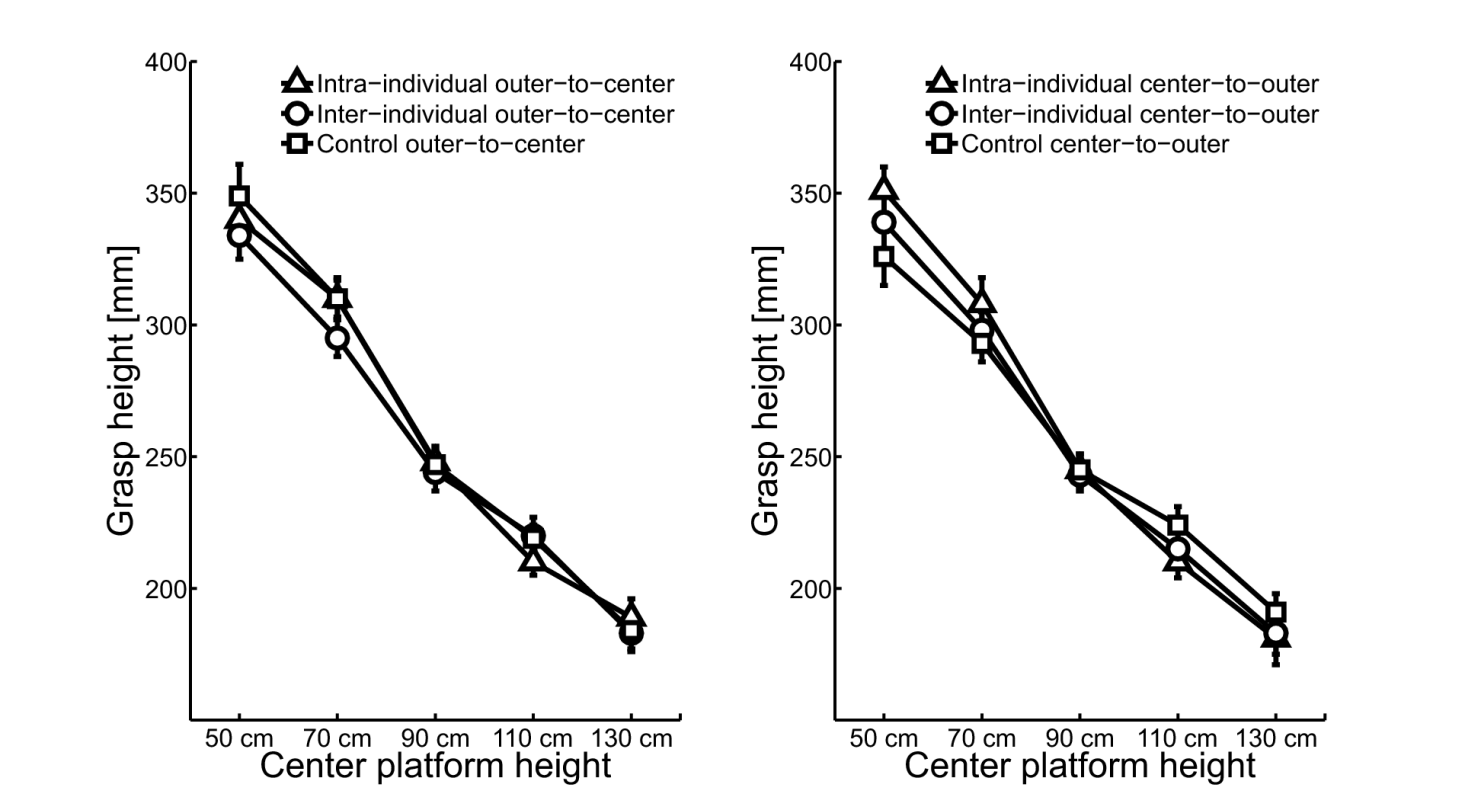
Mean grasp heights (relative to plunger base) as a function of center shelf height during the intra-individual task (triangles), inter-individual task (circles), and control task (squares) for the outer-to-center (panel A) and center-to-outer moves (panel B).

To examine whether the observation of an action is sufficient to elicit recall processes, grasp height data were analyzed using a 2 task condition (intra-, inter-individual) × 2 direction (outer-to-center, center-to-outer) × 2 object position (left, right) × 5 center shelf height (50 cm, 70 cm, 90 cm, 110 cm, 130 cm) repeated measures analysis of variance (RM ANOVA). Analysis indicated that grasp height was inversely related to center shelf height (Figure 2), F(4,92) = 140.86, p <.001, η^2^_p_ = .86. There was no effect of direction or task condition, demonstrating that grasp height was similar for the first and return moves and for the intra- and inter-individual task condition (Figure 2). The slopes for the best-fitting straight lines ranged from -.18 to -.22 and all differed significantly from zero (Table 2).

**Table 2:** Slopes, intercepts, and correlations (r) for best-fitting straight lines relating grasp height (mm) to center shelf height (mm) in outer-to-center and center-to-outer platform moves for each task condition.

		**Outer-to-center**							**Center-to-outer**					
		**Objectposition**							**Objectposition**					
		**Left**			**Right**				**Left**			**Right**		
		**Slope**	**Intercept**	**r**	**Slope**	**Intercept**	**r**		**Slope**	**Intercept**	**r**	**Slope**	**Intercept**	**r**
**Intra-individual**		-.21**	448	0.979	-.20**	434	0.991		-.22**	457	0.986	-.22**	455	0.994
**Inter-individual**		-.19**	432	0.993	-.18***	418	0.995		-.19**	429	0.988	-.20***	440	0.996
**Control**		-.21***	450	0.995	-.21**	454	0.986		-.16***	0.397	0.996	-.17**	418	0.990

The grasp height data of the intra- and the inter-individual task conditions were compared to the control task condition in order to assess potential differences in control priority between variable and fixed platform positions using a 2 task condition (intra-individual, control) x 2 direction of movement (outer-to-center, center-to-outer) x 2 object position (left, right) x 5 center shelf height (50 cm, 70 cm, 90 cm, 110 cm, 130 cm) RM ANOVA. Comparison between intra-individual and control task conditions revealed that the interaction between task condition, direction of movement, and shelf height was significant, F(4,92) = 8.71, p = .001, η^2^_p_ = .28. As the slope of the best-fitting straight lines provide a good and robust estimate of the degree of grasp posture adjustment, we applied the approach of Cohen & Rosenbaum [1] and decomposed the significant three-way interactions based on the steepness of the slopes rather than employing post hoc pair wise comparisons. The slopes of the best-fitting straight lines for the center-to-outer moves in the control task condition were shallower (-.16 and -.17 for object position left and right, respectively) than the slopes for the center-to-outer moves in the intra-individual task condition (- .22 and -.22 for object position left and right, respectively, see Table 2).

Similarly, analysis of differences between the inter-individual and control task condition revealed a significant three-way interaction between the factors task condition, direction of movement, and shelf height, F(4,92) = 4.38, p = .003, η^2^_p_ = .16. Again, the slopes of the best-fitting straight lines for the center-to-outer moves in the control task condition were shallower (-.16 and -.17 for object position left and right, respectively) than the slopes for the center-to-outer moves in the inter-individual task condition (- .19 and -.20 for object position left and right, respectively, see Table 2).

**Figure 3:**
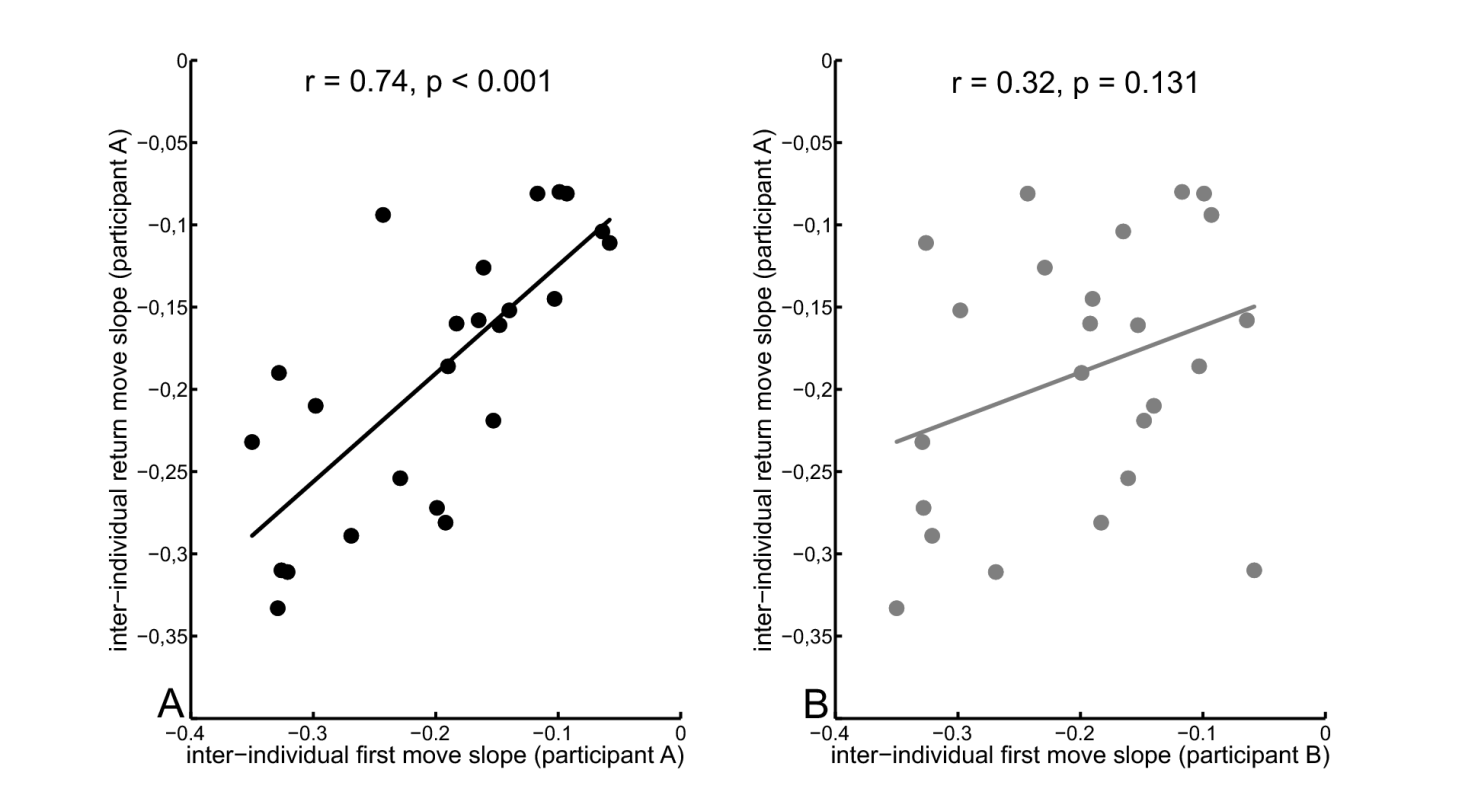
Scatter plot comparing return move slopes of a given participant with first move slopes of the same participant (panel A) and with first move slopes of their partner (panel B) during the inter-individual task condition.

To examine whether participants’ grasp heights of the return moves are more similar compared to their own grasp heights of the first moves or compared to their partners’ first move grasp heights we calculated the slopes for the best-fitting straight lines separately for each participant and direction (first, return) during the inter-individual task condition. Slopes during the return move of a given participant (e.g. A) were highly correlated with the slopes during the first move of the same participant (i.e., A, r = .74, p <.001), but not with the slopes during the first move of their partner (i.e., B, r = .32, p = .131, see Figure 3).

## Discussion

The present experiment addressed two questions. First, we asked whether the observation of an action is sufficient to elicit plan recall processes during a sequential grasp-and-place task. Second, we examined whether a simulated action plan resembles a plan of how the observer would execute that action (i.e., based on the motor representation) or a plan of the actually observed action (i.e., based on the visual representation).

With respect to our first question, our data indicate that the observation of an action is indeed sufficient to elicit plan recall processes. Overall, mean grasp heights for the first move (outer-to-center) were inversely related to center target height during both the intra- and inter-individual task conditions, demonstrating that participants generated an action plan for the first move, and did so to afford more comfortable and controllable body postures when the object was placed onto the center target [1].During return moves, participants grasped the object at a similar height to that used in the first move, indicating that they relied on plan recall [1]. If participants would have generated a new plan for the return move, this should have resulted in similar grasp heights, as in the center-to-outer moves of the control task condition. However, in the control task condition, slopes for the center-to-outer moves were significantly shallower compared to the slopes for the center-to-outer moves, in both the intra- and inter-individual task condition (i.e., the return moves in both tasks).These data argue against the possibility that these findings arose solely because participants attributed greater priority to control at the variable (center) rather than at the fixed (outer) platform heights, and thus support the notion that individuals utilized a plan recall strategy when planning their grasp postures. However it should be noted that participants did not strictly optimize control at the end of the movement when planning their first moves. Rather, grasp heights for the center-to-outer moves in the control task condition also varied as a function of center target height, indicating that some degree of control was also assigned to the start of the movement. These findings are in line with recent results from our laboratory [2,25,26] suggesting that goal-directed planning is guided by a task-specific constraint hierarchy in which specific constraints (e.g. control at the start and at the end of a movement) are weighted relative to each other in order to successfully attain the task goal .

Importantly, grasp heights for the return moves were similar regardless of whether participants had previously performed the first move themselves (intra-individual task condition) or whether they had observed their partner performing that move (inter-individual task condition).These results are congruent with previous research [10,30–32], and suggest that the observation of the first movement triggered an internal simulation of that action plan through the activation of corresponding representations in the observer’s motor system. It is thought that this common neural coding allows the observer to understand other people’s emotions, intentions, and actions [9,30,33],enables the observer to predict the outcomes of the observed actions [8,10,34,35], and allows the observer to extract features from the observed action that can be integrated into their own actions [36–38]. Extending this work, our data provide evidence that a simulated action plan can be held in memory and recalled for subsequent actions. As the generation of a new action plan is associated with cognitive costs [39], relying on plan recall rather than plan generation is a useful strategy to economize on these costs. The inference that can be drawn is that the simulation of an action plan through action observation is associated with similar cognitive costs as the (self-) generation of that plan. Consequently, analogous to recalling a self-generated action plan, reverting to a simulated action plan is an effective strategy to reduce the cognitive burden to the central nervous system.

With regards to the second question, the results of the present study strongly indicate that a simulated action plan resembles a plan of how the observer would execute that action (i.e., based on the motor representation) rather than a plan of the actually observed action (i.e., based on the visual representation). In the inter-individual task, best-fitting slopes during the return move were highly correlated with the slopes during the first move of the same participant, but not with the slopes of their partner’s. Although previous research [40] has shown that recall is based on information stored in extrinsic coordinates (i.e., participants recalled the location where they grasped the plunger rather than the adopted body posture), the results of the present study indicate that participants did not merely observe where their partner grasped the plunger during the first move and then recalled that location and grasped the plunger at the same height for the return move. Our data are also in contrast to recent studies that provided evidence for involuntary imitation in joint action contexts [41-43]. For example, participant dyads in Sacheli et al. [43] grasped bottle-shaped objects as synchronous as possible. Participants were assigned asymmetric roles such that the *Leader* received information about the type of grip used (power vs. precision) whereas the *Follower* was instructed to perform either imitative or complementary actions. Results showed that *Followers* tended to imitate their partner even in the complementary action context where imitation is detrimental to joint performance.

Rather, our data suggests that observing the action triggered a simulation of that action by activating the observer’s specific motor representation of that action (i.e., how the observer would perform that action) which can be kept in memory and used for subsequent actions. These findings are in accordance with studies who argue in favor of more flexible models of perception-action coupling in where the action context takes in a critical role in determining the relationship between action observation and action execution [44-46]. Participants in the study of van Schie et al. [46], for example, initiated identical actions faster in an imitative context but non-identical actions faster in a complementary context suggesting that participants were able to inhibit the tendency to imitate the observed action.

Methodological differences between these studies might account for the apparent divergent results. In the studies that provided evidence for involuntary imitation [41-43], participants performed synchronous actions embedded in realistic interaction scenarios (i.e., together with a real human partner). In contrast, previous studies that argue against the automatic nature of imitation [44-46] employed only joint-like interactions in where participants observed their partner displayed on a computer monitor and responded accordingly. Given that we employed a realistic interaction scenario, one possibility for the absence of imitation effects in the present study might be substantiated in the sequential character of our task (i.e., one participant completed the movement before the partner commenced his or her movement). Consequently, the time delay between observed and (self-) executed action might allow the observer to inhibit to act in a mimicking fashion.

Alternatively, unequal biomechanical costs associated with the different movements might have contributed to the deviating results between the present study and the study of Sacheli et al. [43]. That is to say, in Sacheli et al. [43], the tendency to imitate the partner’s motor action was primarily evidenced by a change in maximum wrist height which, we argue, would not likely result in substantial increases in biomechanical costs. In contrast, if participants in the present study were prone to imitate their partner (i.e., grasp the plunger at the same height during the return move as their partner during the first move), they might have had to adopt uncomfortable and biomechanically costly grasp postures. Consequently, simulating actions based on one’s own specific motor representation is biomechanically and cognitively advantageous and strengthens the existence of a cognitive system that takes into account a person’s physical competencies when interacting with the physical world [32].

Together, our results not only provide further evidence that participants mentally simulate observed actions, but that these simulated action plans are recalled and used for subsequent own actions. Furthermore, a simulated action plan is likely to resemble a plan based on the observer’s specific motor representation rather than a plan of the actually observed action performed by the interaction partner which provides further evidence for a flexible mechanism of perception-action coupling.
